# Compatible Photochromic Systems for Opto-electronic Applications

**DOI:** 10.1021/acs.jpcb.1c08728

**Published:** 2021-12-04

**Authors:** Adam Szukalski, Aleksandra Korbut, Karolina Zieniewicz, Sonia Zielińska

**Affiliations:** † Faculty of Chemistry, Advanced Materials Engineering and Modelling Group, Wroclaw University of Science and Technology, Wybrzeze Wyspianskiego 27, Wroclaw 50370, Poland; ‡ Faculty of Chemistry, Department of Polymer Engineering and Technology, Wroclaw University of Science and Technology, Wybrzeze Wyspianskiego 27, Wroclaw 50370, Poland

## Abstract

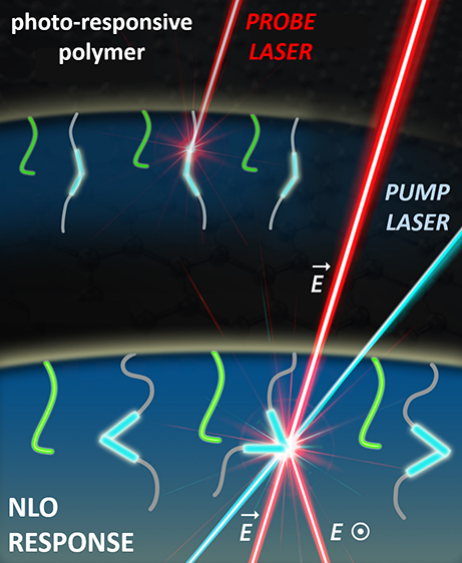

Today, a lot of attention is paid to remote controlled opto-electronic devices. Many of them are commonly used in the society, industry, and science. Accessories dedicated to the particular utilization are desired. The point is to find a simple way to obtain smart and functional appliances. Materials engineering faces such problems and provides a variety of solutions concerning advanced material design, preparation, and utilization. Photochromic materials represent one of the already known materials, which still find other objectives in new fields of life. In our work, we present two differently constructed photoresponsive polymers, which give significantly different nonlinear optical (NLO) response visible as noticeable optical signal modulation. By playing with diversified laser light energy or its frequency, NLO output characterized appealing, and individual characteristics (doubled Δ*n* ∼0.02 vs 0.04 and entirely different kinetics for two similar materials and the same laser pumping). Interestingly, high output signal repeatability and stability were achieved, which indicate the investigated materials as promising candidates in the construction of various opto-electronic devices. Additionally, a set of photoresponsive studies, reflectometry, and theoretical insights was performed and included in this work.

## 1 Introduction

Currently, plenty of research groups are focused on organic, multifunctional, smart materials. Many of them connect various interesting attributes like optical properties together with the possibility of electro-optical modulations or electric/mechanical control.^1^ Light characterizes the most valuable advantage, which is signal duration transmission. Indeed, photons constitute the most rapid information carrier. When considering remote controlled systems, such parameter is crucial. This is the reason why advanced materials utilized in spectroscopy, photonics, and opto-electronics possess superiority than other so-called ″cable systems″ (obviously, excluding waveguides).^2,3^ For this reason, if considering optical networks or singular computer units, it is understandable to have the fastest systems for data administration. Photoresponsive organic systems can serve as the efficient, repeatable, and stable logic gates necessary in reconfigurable networks or optically based systems.^4−6^ All-optical switching, discovered many decades ago by John Kerr,^4,7^ perfectly fits the challenges given by the 21st century. Few variants of the Kerr effect are known: the Kerr (quadratic electro-optic; QEO) effect, optical Kerr effect (OKE is also known as optical-optic or all-optical or AC Kerr effect), and magneto-optic Kerr effect.^7^ If considering the OKE phenomenon, the electric field (energy) is provided to the illuminated medium from the electromagnetic wave (light). Such approach uses the fastest energy/information carrier and fulfills all theoretical basics related to the energy transfer, intermolecular interaction, and influence. Basically, only one light (laser) source is enough to manipulate an isotropic material and convert it to an efficient NLO medium; however, for the needs and clarity of the experiment, the second laser line is utilized to monitor the whole process.^7^

Photochromic polymers easily meet the afore-mentioned demands. Many of them were already considered in holography, or at least in its most basic form, namely, (surface) relief gratings,^8−10^ or other light-responsive systems serving as sensors,^11^ carriers,^12,13^ or membranes.^14^ Based on the same optical Kerr effect, optical switches^15,16^ creating more sophisticated, integrated optical networks^17,18^ were implemented. However, independently of the already achieved and published polymeric systems, still a lot of issues need optimization or precise individualization. It can be stated that dedicated or intentionally designed solutions can cooperate much more efficiently than generally produced and introduced systems. For instance, an optical switch dedicated to previously selected laser light sources (together with their power, working time regime, and modulation frequency) can be easily optimized to the photoresponsive (co)polymer, which will operate in a defined current or voltage range.^19,20^ Not only light controlled features can be devoted. Other examples take into account the following features: thermal stability,^21^ flexibility,^22^ polymer mass, and/or glass transition value.^23^

Here, we present two new co-polymers consisting of the same main chain and the same photoactive side chains but differ in various photopassive side groups. As the photosensitive fragment, the azobenzene structure plays a pivotal role, whereas as the nonactive hindrance, two groups were utilized: long aliphatic chain (-SMA) and compressed moiety (-AA), respectively. Both of the co-polymers gave a nonlinear optical response due to the azo-fragment, which is light sensitive and undergoes photoinduced molecular transformation (trans → cis → trans). Besides typical investigations, like reversible conformational change distribution, an additional technique—reflectometry—was applied. Subsequently, to achieve a deeper insight into investigated molecular structures, a set of quantum chemical calculations was done. Finally, third-order NLO properties were investigated to characterize the nonlinear optical response of the investigated materials. Since the comprehensive studies were done considering new materials, starting from the synthesis route and its details, an extensive collection of the estimated parameters of the materials *sensu stricto* (^1^H NMR, *T_g_*, ρ, *C_p_*) and the spectroscopic (α, *n*), kinetic (i.e., τ_inc_^stat^ and τ_dec_^stat^), and NLO (Δ*n*, *n*_2_, χ^(3)^, β) constants was presented. Achieved experimental developments constitute proof of the undoubted importance of materials engineering in the context of the construction of new-generation functional photoresponsive polymers.

## 2 Materials and Methods

### 2.1 Synthesis

Azo dye (SMERe) and monomer M-SMERe were synthesized as reported previously.^6^ The coupling reaction of the diazonium salt of sulfamerazine with 2-(*N*-ethylanilino)ethanol was used to form the azo dye. Consequently, a reaction of the dye with methacrylic anhydride was performed to obtain a methacrylic monomer. Random co-polymers p(SMERe-AA) and p(SMERe-SMA) were synthesized by free radical polymerization using equimolar amounts of monomers and AIBN as an initiator. The amounts of co-monomers and solvents, reaction conditions, and yields of the resulting co-polymers are summarized in Table S1 in the Supporting Information.

### 2.2 Quantum Chemical Calculations

For quantum chemical calculations, the Gaussian 16 software was used.^24^ The geometry of the repeating units occurring in polymers described in this work was optimized using the RHF method and 6-31g basis set. The same approach was implemented to calculate the first hyperpolarizability of the azobenzene-based molecules, characterizing their nonlinear optical properties. That combination brought reasonable results in our previous works.^25^

### 2.3 Spectroscopy

#### 2.3.1 UV–Vis

Absorption spectra were recorded on a Hitachi U-1900 spectrophotometer. The measurements were carried out before and after irradiation with a laser beam at 445 nm wavelength for various exposure times. UV–vis experiments were performed for the thin films prepared by the spin-coating technique using Laurell’s WS-400-B-NPP-LITE spin coater. The spin up speed was set at 1200 rpm for 20 s. Thin films were prepared from solutions of co-polymers p(SMERe-AA) and p(SMERe-SMA) (5 wt % in THF or 5 wt % in chloroform, respectively) filtered through a syringe filter. After deposition on the glass plate, the films were dried at 50 °C for 24 h.

#### 2.3.2 Reflectometry

The thin film analyzer Filmetrics F20 was used to estimate the refractive index value. Co-polymer solutions were deposited on a silicon wafer using the spin-coating technique. Measurements were conducted before and after 5 min of laser beam irradiation (λ = 445 nm).

#### 2.3.3 Nonlinear Optics

**Figure 1:**
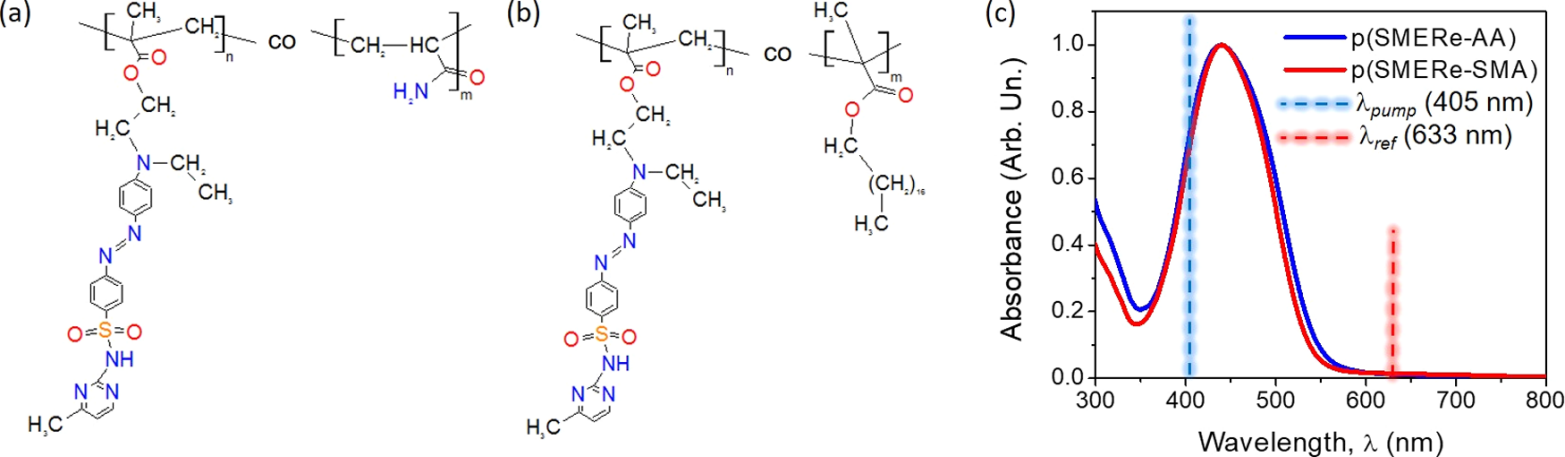
Chemical structure of the investigated photochromic polymers p(SMERe-AA) (a) and p(SMERe-SMA) (b) and their absorption spectra (c), respectively. The reference and pump laser beams utilized in all-optical switching investigations were marked in dashed red and blue lines, accordingly.

The all-optical switching phenomenon in the considered polymeric materials was investigated according to the principles of the optical Kerr effect, which was already described before elsewhere.^7,25,26^ Briefly, to control and induce the NLO effect, two laser sources are needed. One of the laser line serves as the reference (or control) beam, and from theory, its wavelength should be out of the absorption resonance of the investigated material. The second laser source has to be absorbed by the photoactive material to interact with such kind of supplied energy. The latter laser is called ″pump″ or ″inducing″ beam. So, the all-optical switching phenomenon can be easily investigated using a typical *pump-probe* setup, which was schematically shown in the literature before.^7,25,26^ Two laser lines, which were implemented in the considered experiment, were marked as blue (pump beam) and red (reference line) in Figure 1c. To achieve and measure the NLO signal, the sample was placed into a cross-polarizer system, and the output beam was collected by a photodiode. Any aberrations were excluded thanks to the UV cutoff filter mounted just before the opto-electronic collecting device. Since both of the implemented laser lines irradiate on the same spot on the sample surface, the NLO phenomenon starts only at that moment. If any of the pump beam stops or in the presence of thermodynamic conditions (including the reference laser illumination), no effect is observed (no output signal). To observe static (or total) photoinduced birefringence in the photochromic polymers, the pump laser beam has to be provided for a long time (from seconds up to hours),^7,25,26^ whereas dynamic Δ*n* changes (conformational transformations) can be acquired when the pump laser beam is modulated (i.e., by a mechanical chopper, in the frequency regime of 10–500 Hz).

To estimate the Δ*n* value, the following equation can be used:^7,25,26^
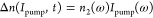
1where *n*_2_ and *I*_pump_ denote the second, nonlinear optical refractive index values and pump beam intensity, both related to the ω frequency, respectively. However, the straightforward relation between photoinduced birefringence vs experimental setup configuration can be found in another theoretical relation:^7,26^
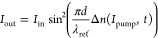
2where *I*_out_ and *I*_in_ refer to the output and initial reference laser intensity, respectively; *d* denotes the active layer thickness; and λ_ref_ means the reference laser wavelength in nm (in our case, it is equal to 632.8 nm).

Finally, the third-order nonlinear optical susceptibility value (χ^(3)^) considered in the SI unit system can be estimated with the following equation:^7,26^
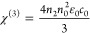
3where *n*_0_ denotes the value of the linear refractive index of the investigated system and ε_0_ and *c*_0_ mean the dielectric constant and light speed in a vacuum, respectively.

## 3 Results and Discussion

### 3.1 Synthesis Route and Product Characterization

Free radical polymerization was used to synthesize two photochromic polymers: p(SMERe-AA) and p(SMERe-SMA). The reaction was carried out in a round-bottom flask with 10 wt % AIBN relative to both co-monomers. The chemical structure of azopolymers is illustrated in Figure 1. The resultant co-polymer structures were precisely characterized and confirmed (including their chemical purity) using ^1^H NMR spectroscopy. ^1^H NMR spectra of both co-polymers showed signals characteristic for protons at two double-substituted benzene rings and a pyrimidine ring (∼6.90–8.15 ppm). Moreover, on the spectra were also recorded signals from the methyl group in the pyrimidine ring (∼2.60 ppm) and broad multiplets from protons of methylene groups in the main chain of the polymer. On the ^1^H NMR spectrum of p(SMERe-SMA), multiplets from protons of methylene groups of the aliphatic long chain at ∼1.10–1.25 ppm were also observed. On the spectra of both co-polymers, no signals corresponding to protons at carbon atoms in the double bond were observed. It confirmed that monomers were reacted completely.

Based on the ^1^H NMR spectra, the content of the azo part (%_AZO_) in the co-polymer was estimated on the integrals using the following equation:^27^
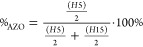
4where H5 and H15 (or H9 and H19 for p(SMERe-SMA)) are integrals of the signals at ∼8.15 and 1.49 (or ∼0.81) ppm, respectively. The calculated value of the final molar content of the azo part was similar for both investigated co-polymers: 48 mol % for p(SMERe-AA) and 49 mol % for p(SMERe-SMA). The ratio of AZO/AA or AZO/SMA in the synthesized polymers was close to the theoretical value (co-monomers utilized in polymerization were used in a 1/1 mol/mol ratio). Complete ^1^H NMR spectra and their description are presented in the Supporting Information (Figures S1 and S2).

Basic properties of the synthesized novel co-polymers are given in Table 1. The average molecular weights determined by GPC indicate that resulted co-polymers were oligomers, consistent with other experimental data reported for similar materials in the literature.^28,29^ This may be associated with the addition of a large amount of the AIBN component during the free radical polymerization.^30,31^ On the other hand, decreasing the amount of the initiator could significantly reduce the efficiency of the reaction. The type of nonchromophoric co-monomer enables one to modify the physical properties, e.g., the glass transition parameter. It can be observed that the co-polymer that contains stearyl methacrylate as the co-monomer (p(SMERe-SMA)) has a much lower value of *T_g_* compared with the p(SMERe-AA) co-polymer.

**Table 1:** Material Features of the Synthesized Co-polymers

	properties						
polymer	*M*_n_ (g/mol)	*M*_w_ (g/mol)	*M*_w_/*M*_n_	*T*_g_ (°C)	*n*_r_	Δ*n*_r_	λ_max_^ABS^ (nm)
p(SMERe-AA)	3600	12,000	3.3	113	1.568	0.0149	436
p(SMERe-SMA)	1400	4400	3.1	78	1.585	0.0143	438

### 3.2 Theoretical Insights into Material Properties

Geometry optimization at the RHF/6-31g level of theory was performed for the trans and cis isomers of the azobenzene-containing SMERe repeating unit but also for SMERe-acrylamide dimers as well as for SMERe-octadecyl methacrylate dimers in both conformational forms. Basic properties derived from the geometry optimization results (including dipole electric field polarizability and hyperpolarizability calculation) are gathered in Table 2.

**Table 2:** Basic Properties of the Trans and Cis Isomers of SMERe Repeating Unit and Its Dimers

compound acronym	spatial form	Δ*E* (kJ/mol)	*V*_m_ (cm^3^/mol)	*a*_0_ for SCRF (*Å*)	μ (D)	α × 10^39^^a^ (C^2^m^2^/J)	β × 10^46^^b^ (C^3^m^3^/J^2^)
SMERe	trans	65.1	308.1	5.96	10.2	3.57	3.51
	cis		385.8	6.39	14.0	3.01	0.16
SMERe dimer	trans	83.0	633.6	7.44	21.7	7.15	5.78
	cis		672.9	7.59	12.3	7.15	4.25
SMERe-SMA dimer	trans	75.1	675.4	7.59	12.2	5.11	4.20
	cis		614.5	7.37	14.1	5.22	0.21
SMERe-AA dimer	trans	37.1	421.5	6.56	12.1	3.87	3.96
	cis		349.6	6.20	6.18	6.62	1.58

a Polarizability.

b Hyperpolarizability; both values calculated with the Gaussian software for two isomers, trans (E) and cis (Z), respectively.

The calculated values of the dipole moment, molar volume, polarizability, and first hyperpolarizability parameters were similar for all compounds. It is assumed that these originate from the azobenzene derivative unit, which is the same for all considered model compounds. The most distinct differences between the trans and cis spatial conformers were distinguished in the case of the dipole moment and first hyperpolarizability values. Furthermore, the calculated β values are higher for the trans isomers, while in the case of α and μ, there is no unambiguous dependence. According to calculated results, the photoisomerization transformation from trans to cis forms of the chromophore led to a distinct increase of the calculated molar volume in most cases. Geometry optimization of different model compounds representing selected possible polymers chains led us to find the possible hydrogen bond formation upon the trans → cis isomerization for the acrylamide-containing co-polymers (Figure S3). The formation of the hydrogen bonds between the −NH_2_ group in acrylamide repeating units and −SO_2_ in the neighboring SMERe repeating unit seems to be privileged sterically upon isomerization toward the cis form. That kind of interaction may be responsible for the stabilization of the cis isomers and decrease in the relaxation rate and efficiency. Basic properties of both spatial isomers of SMERe, and its co-polymers with acrylamide and stearyl methacrylate, are presented in Table 2.

Additionally, the physicochemical properties calculated using the Synthia module implemented in the Materials Studio package are presented in Table 3. The Synthia module calculates polymer properties using advanced quantitative structure–property relationships. It allows us to rapidly screen polymer models for a wide range of properties. The calculation algorithm uses topological information and connectivity indices derived from the graph theory, so it is essentially based upon individual atoms and bonds. The complex theoretical background of that calculation methodology is presented in the book of Bicerano.^32^ Such screening opportunity may help to improve the new material design process.

**Table 3:** Theoretical Values of the Physicochemical Properties of Homopolymer p(SMERe) and Co-polymers (p(SMERe-AA) and p(SMERe-SMA))

compound acronym	*M*_w_	*T*_g_ (K)	α^a^	*V*_m_ at 298 K	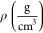	*C*_p_ of the solid	*n*	*E* (GPa)
p(SMERe)	5	333	290	402	1.26	605	1.583	8.5
	10	349	277					8.8
	15	360	270					9.0
	∞	408						
p(SMERe-SMA)	5	273	689	374	1.12	567	1.529	5.9
	10	287	661					5.6
	15	296	646					5.6
	∞	325						
p(SMERe-AA)	5	342	282	229	1.26	351	1.576	6.6
	10	361	269					6.8
	15	371	262					7.0
	∞	404						

a Coefficient of volumetric thermal expansion.

Properties were determined for the following assumptions: environmental temperature of 298 K and the value of molecular weights of homo- and co-polymers both equal to 5000, 10,000, and 15,000 amu, respectively.

Calculated values of the refractive index seem to be in good agreement with those derived experimentally by the reflectometry technique. For p(SMERe-AA), the theoretical value of the *n* parameter is overestimated only by 0.5%. The *T*_g_ estimation of co-polymers was less accurate. The model of p(SMERe-SMA) proved to be the most elastic, which is consistent with optical measurement results. The glass transition temperature was comparable for homopolymer p(SMERe) and p(SMERe-AA) and significantly lower for p(SMERe-SMA), while the molar volume calculated with Synthia was found to be comparable for p(SMERe) and p(SMERe-SMA) and significantly lower for p(SMERe-AA). The coefficient of volumetric thermal expansion is significantly higher for the p(SMERe-SMA) co-polymer than for other materials.

### 3.3 UV–Vis Spectroscopy and Reflectometry

**Figure 2:**
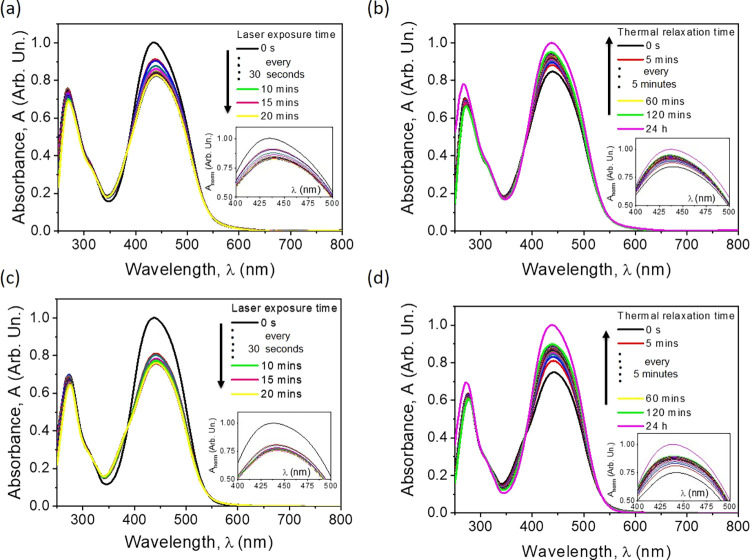
Absorption spectra kinetics during the trans*–*cis photoisomerization reaction induced by the UV laser light (a, c) and also when the cis*–*trans reverse process (thermal relaxation) in the dark conditions is observed (b, d) for p(SMERe-AA) (a, b) and p(SMERe-SMA) (c, d), respectively.

The photoisomerization phenomenon of both co-polymers was investigated in the form of thin films by laser light irradiation (λ at 445 nm). To ensure that all of the azobenzene units sustained in the lower in energy, trans molecular configuration, the azopolymer films were stored in the dark at room temperature for 1 day before the measurements. Afterward, the reversible trans*–*cis*–*trans transformation kinetics were examined using UV–vis spectroscopy. In that way, it was possible to record the process of photoisomerization induced by the laser light and then the thermodynamic relaxation without any stimuli. Figure 2 shows the effect of UV illumination and the thermal recovery of the investigated materials. The maximum value of the absorption band position (λ_max_) of the considered co-polymers was observed in the blue region of the spectrum. The ones localized in the range of 436–438 nm were assigned to the characteristic π–π* and *n*–π* transitions of the azobenzene derivatives.^33^ Along with the UV irradiation time, the π–π* band intensity decreased rapidly, whereas the *n*–π* absorption band for the cis isomers at ∼375 nm was noticed. It suggests that the trans isomers of the azobenzene derivative transformed into cis forms under the UV laser illumination, and therefore, a thermodynamic equilibrium state was reached. With reference to Figure 2, two isosbestic points were distinguished at ∼385 and ∼540 nm, respectively. Subsequently, after switching off the UV light source, the thermal relaxation process was carried out in the dark conditions when no external stimuli were applied. Thus, the phototransformation back reaction was slow, and the value of absorbance around 436–438 nm increased. Interestingly, the reversibility of the conformational change reaction was achieved and experimentally proven. A similar behavior was observed for p(SMERe-AA) and p(SMERe-SMA) co-polymers, respectively.

Kinetics of the trans*–*cis photoisomerization of considered polymers were analyzed according to the following equation:^34^
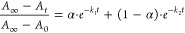
5where *A*_0_, *A_t_*, and *A_∞_* represent absorbance intensity values of the trans form corresponding to the time 0, *t*, and infinite, respectively. The α coefficient describes a fraction of the fast photoisomerization stage in the total system’s conversion. Subsequently, *k*_1_ and *k*_2_ are the rate constants of the trans*–*cis conformational transformation. The trans-to-cis photoinduced transformation rate constant (*k*_1_) of p(SMERe-AA) was found to be much higher than that of p(SMERe-SMA), i.e., equal to 87.0 × 10^–3^ and 23.5 × 10^–3^ s^–1^ (Table 4), respectively. Due to the large size of the long side-chain aliphatic of the stearyl methacrylate co-monomer, the movements of the azobenzene groups in the polymer chain were significantly limited. In effect, the chromophore group in stearyl methacrylate co-polymers had less freedom to move during the isomerization cycle. Indisputably, the mobility of derivatives containing azo-moieties in the side-polymer chains strongly depends on the free volume distribution around the chromophores.^35^

**Table 4:** Kinetic Parameters of the Trans-to-Cis Photoisomerization and Reverse Cis-to-Trans Dark Thermal Relaxation of Co-polymers Determined by UV–Vis Measurements

	polymer	
kinetic parameters	p(SMERe-AA)	p(SMERe-SMA)
Photoinduced trans → cis transformation		
*A*_∞_/*A*_0_	0.83	0.76
α	0.52	0.63
*k*_1_ × 10^–3^ (s^–1^)	87.0	23.5
1 – α (−)	0.48	0.37
*k*_2_ × 10^–3^ (s^–1^)	7.95	9.55
Dark, thermal relaxation (cis → trans)		
α’	0.35	0.34
*k*_1_’ × 10^–3^ (s^–1^)	6.38	14.73
1 – α’ (−)	0.65	0.66
*k*_2_’ × 10^–3^ (s^–1^)	0.089	0.105

Hereupon, the reverse cis*-*to-trans isomerization in the darkness was also investigated. As shown in Figure 2, the cis isomers slowly returned to the original and thermodynamically more stable trans forms. Along with the relaxation time, the absorption band localized at ∼436–438 nm increased in intensity to the initial state value before UV laser irradiation. The relaxation process was carried out for 24 h. The thermal relaxation kinetics can be fitted to the following equation:^34^
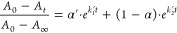
6where *k′*_1_ and *k′*_2_ denote rate constants related to the cis-to-trans reverse isomerization and α*′* describes the fraction in the total conversion of the system. The cis*–*trans rate constants (*k′*_1_ and *k′*_2_) were significantly different compared to the trans*–*cis rate constant values. Moreover, the *k′*_1_ rate constant for co-polymer p(SMERe-SMA) was more than twice as the same parameter for p(SMERe-AA), i.e., 14.73 × 10^–3^ and 6.38 × 10^–3^ s^–1^, respectively. The back reaction process in both co-polymers films was significantly slower than the photoinduced trans*–*cis isomerization. Such behavior can be explained by the process environment (stimulus) causing the thermal relaxation, which was conducted in the dark without any laser light irradiation. This fact seems to be in agreement with experimental data obtained for the other azopolymers described in the literature.^35^ These results confirmed that investigated azo-co-polymers undergo the very well-known reversible conformational transformation (trans*–*cis*–*trans multiple isomerization). The plots of photoisomerization kinetics are shown in Figure S4 in the Supporting Information.

Additionally, the photochromic properties of analyzed co-polymers were also investigated using the reflectometry technique, which serves for the refractive index value determination. It was estimated from the ratio of reflectance coefficients vs the sample surface. From the reflectometry measurements, the real part of the refractive index value (*n*_r_) was calculated as 1.568 and 1.585 for p(SMERe-AA), and p(SMERe-SMA), respectively. Then, the thin films were illuminated with the same UV laser light, which was used before. After 5 min of irradiation, the measurements were repeated to define changes of the refractive value (Δ*n*_r_). For both investigated co-polymers, similar results were obtained, i.e., Δ*n*_r_ ∼0.014.

### 3.4 All-Optical Switching in Photochromic Polymers

**Figure 3:**
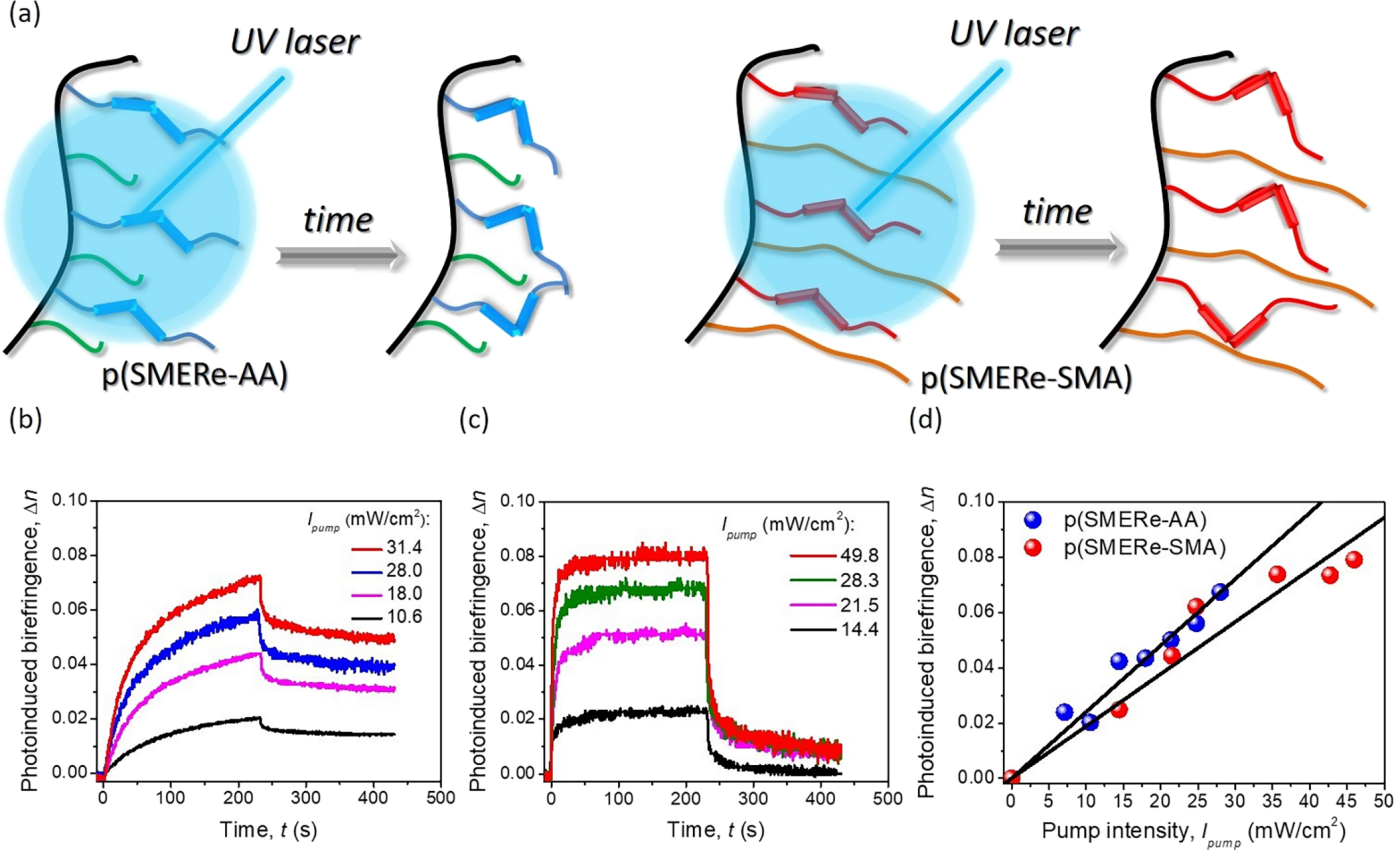
Scheme of photoinduced remote control of refractive index changes (a); optical birefringence kinetics generated by various pump beam intensities for p(SMERe-AA) (b) and p(SMERe-SMA) (c); and Δ*n* vs *I*_pump_ correlation (d).

The all-optical switching phenomenon in the considered photochromic polymers is schematically shown in Figure 3a. Light driven structural changes were provided by UV laser light irradiation, which affected straightforwardly the azobenzene fragments localized in the side chains (marked in blue and red strips for p(SMERe-AA) and p(SMERe-SMA), respectively). Such delivered and suitable photon interaction with an active NLO material leads to the molecular reorientation over time following the multiple trans*–*cis*–*trans phototransformations. The process continues as long as any of the azobenzene fragments still absorb the UV light according to eq 2. Consequently, the photostationary state is achieved and the molecular population (as much as possible limited just by the free volume surrounding photoactive fragments) is ordered, making an organic system optically anisotropic due to the refractive index indicatrix shape. The kinetics of the aforementioned photophysical process are shown in Figure 3b,c for both photochromic polymers: p(SMERe-AA) and p(SMERe-SMA), respectively. As it is presented for various (increasing) UV light beam intensities in the first case, a phototransformation process is rather one-directional because the reversibility level is low (ca. 30%). However, the maximum achieved value of the photoinduced birefringence (when *I*_pump_ ∼31 mW/cm^2^) was observed to be around 0.07, which is a significant change, whereas in the case of the p(SMERe-SMA) organic system, the Δ*n* modulation is bidirectional with almost 100% reversibility (Figure 3c). The highest achieved NLO response is similar as before and is equal to ca. 0.08. Interestingly, if considering the p(SMERe-AA) system, the photostationary state (*plateau* range) after ∼250 s of UV laser light irradiation was not achieved and the light controlled ordering process seemed to be slow and less efficient than that in the other case. The p(SMERe-SMA) NLO organic system gives fast Δ*n* saturation, which is visible as the *plateau* range of the experimental curves after less than 50 s. What needs to be underlined is the same photoactive side-chain construction for both photochromic polymers. They differ in nonactive side chains due to both the used atoms and their numbers (cf. Figure 1a,b). The acrylic group in the p(SMERe-AA) polymer is expanded just by the amine moiety, while in the p(SMERe-SMA) NLO system, a long aliphatic chain is attached. In the first mentioned co-polymer, the nonactive side chains do not cause a significant spatial hindrance for the photoactive azobenzene fragments. The other polymeric chains with their active parts can easily intercalate from the adjacent molecules (due to the hydrogen bonding presence). Therefore, it causes the only one spatial obstacle for the photoisomerization processes, which can be randomly interrupted. Hypothetically, for this reason, the photoinduced birefringence kinetics in this case are slower and slightly less effective. If looking at the p(SMERe-SMA) NLO system construction, the nonactive side chains can provide small rotations due to the sp^3^ hybridization of the carbon atoms presented there. Therefore, the long aliphatic chains create some order between active and nonactive side groups in the considered polymer. Then, since the free volume is provided close to the azobenzene fragments, the phototransformation processes can take place fast and efficiently. From that reason, the only difference observed during all-optical switching phenomenon investigation is the kinetics (significantly diverse), although not the Δ*n* efficiency (slightly divergent) (Figure 3). The linear increase of the photoinduced birefringence vs pump beam intensity is shown in Figure 3d, which confirms the third-order NLO character of the performed experiment.^7^

**Figure 4:**
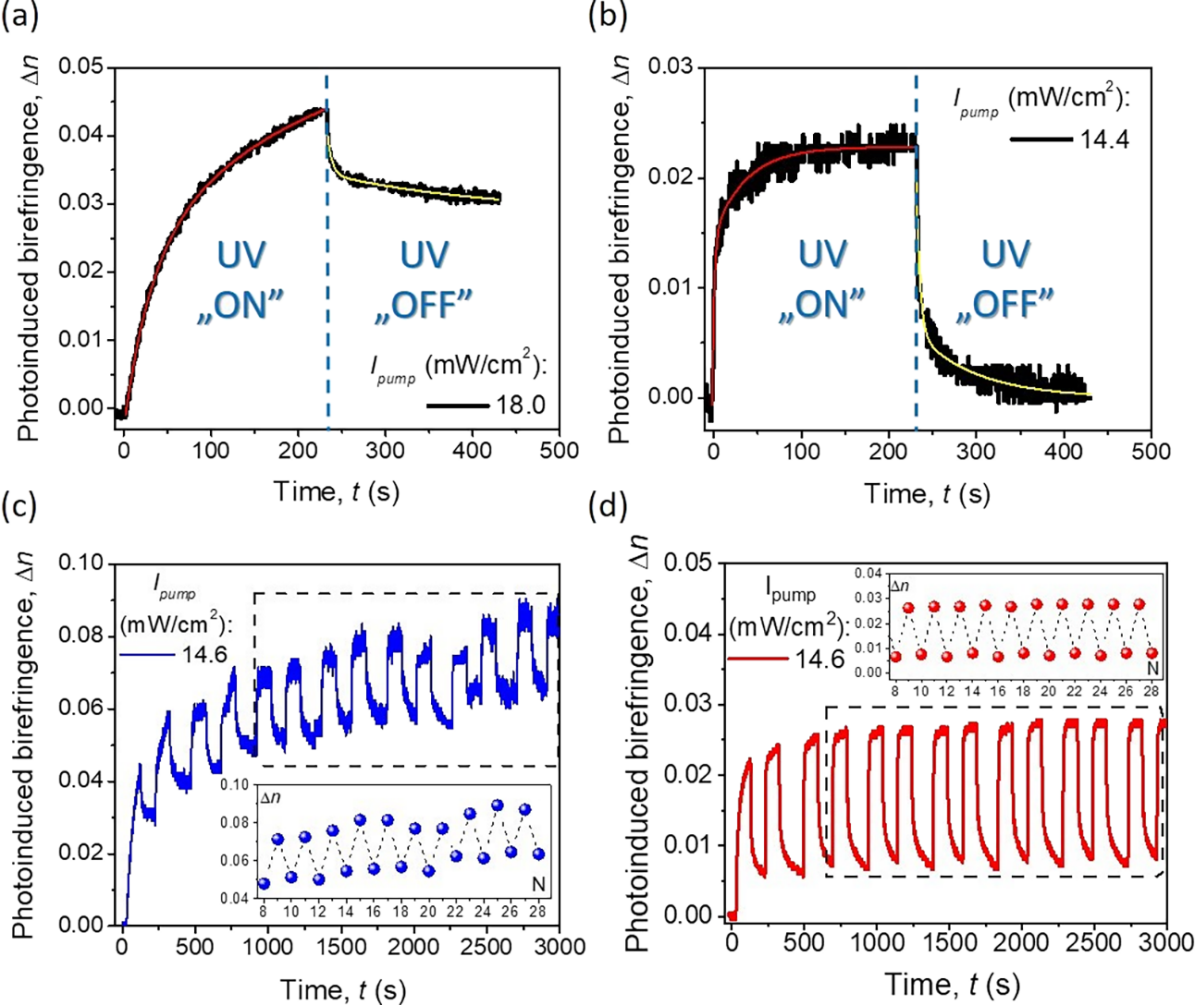
Δ*n* kinetics approximation using biexponential functions marked in red and yellow when laser is on and off (a, b), respectively; multiple optical switchings of the photochromic NLO systems (c, d) for p(SMER-AA) and p(SMERe-SMA), respectively.

The further analysis of the photoinduced birefringence kinetics is presented in Figure 4. The Δ*n* signal increase and thermodynamic decay were approximated using biexponential functions according to the assumptions presented in the literature elsewhere.^36−38^ Such approach includes both photoactive azobenzene fragments (light ones) and macromolecular chain movements (heavy parts) leading to the UV-light controlled photoalignment. The time constants describing the kinetics of the UV-induced molecular reorientations are equal to τ_1_^inc^= 30.6 ± 0.8 s and τ_2_^inc^= 222.7 ± 20.0 s for signal increase and τ_1_^dec^= 6.3 ± 0.3 s and τ_2_^dec^= 373.0 ± 114.7 s for signal decay in the p(SMER-AA) material, respectively (Figure 4a). Meanwhile, the p(SMER-SMA) co-polymer is defined by τ_1_^inc^= 2.3 ± 0.1 s and τ_2_^inc^= 37.1 ± 0.3 s for light-controlled signal increase and τ_1_^dec^= 4.2 ± 0.2 s and τ_2_^dec^= 67.9 ± 5.4 s for thermodynamic signal decay, respectively (Figure 4b). As expected, the first coefficient values in abovementioned polymers are much smaller than the second ones, which straightforwardly result from the photoinduced reorientation type (azobenzene fragments vs polymeric chains). Interestingly, the p(SMER-SMA) polymer seems to achieve a much faster photostationary state (*plateau* range after ca. 150 s of UV irradiation, Figure 4b). As clearly seen, the molecular reorientation dynamics are much higher (by about 1 order of magnitude) for the p(SMERe-SMA) polymer than for its equivalent, namely, p(SMERe-AA). Our hypothesis leads to their chemical structure differences, which in the first case is more ordered (thanks to the long aliphatic chain having a comb-like structure) and in that way sustains the free molecular volume needed for light induced phototransformations. This is the reason for the much faster molecular reordering creating optical anisotropy in the p(SMERe-SMA) polymer. From the same structural reason, namely, small acrylamide group, which does not constitute spatial hindrance, it causes higher lability for the neighboring photoresponsive groups, which can use the available free space for random, energetically controlled movements, making in that way higher entropy. The polymer chain entanglement takes place, which can require a higher energy (or time) to provide the same photoreorientation effect, as in the case of p(SMERe-SMA).

Then, multiple NLO signal recordings fully controlled by UV light were collected for both polymers (Figure 4c,d). The previously presented conclusions found attestation in numerous UV laser light switchings. In the case of the p(SMER-AA) polymer (characterizing a more chaotic chemical structure with higher entropy), the achieved NLO response is slower and still slightly increasing toward a photostationary state. Collected amplitude (shown in the Figure 4c inset) seems to be rather stable and is equal to ∼0.02 of the Δ*n* signal. Also here, even if the used pump beam intensity was the same like in the next case, the photoinduced birefringence characterizes higher values. Going to the p(SMER-SMA) co-polymer, it seems that after very few cycles of switching on/off the incident UV light, the nonlinear optical response is on the same, very stable level and provides highly repeatable NLO signal modulation (amplitude is again estimated to be about 0.02 of Δ*n*). The series of imposingly symmetrical and repetitive NLO response modulation provided by the fully organic photochromic material is shown in the inset of Figure 4d.

**Figure 5:**
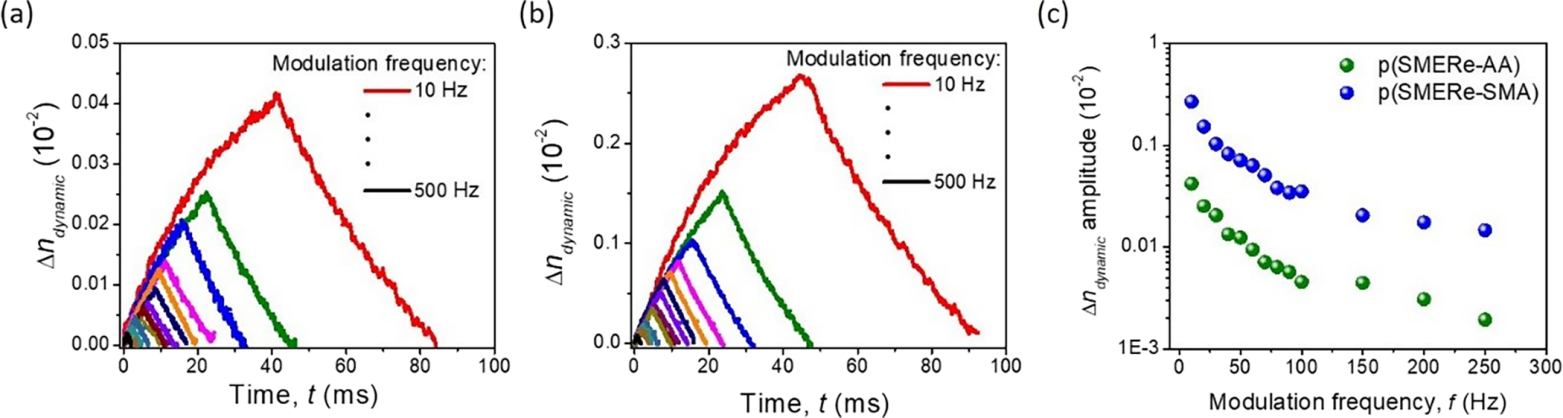
Kinetics of the dynamic part of photoinduced birefringence (trans*–*cis conformational changes) for p(SMERe-AA) (a) and p(SMERe-SMA) (b), respectively; Δ*n* vs frequency modulation of the pump beam signal (c). *I*_pump_: 14.4 mW/cm^2^.

The so-called dynamic OKE signal represents molecular trans*–*cis–trans phototransformations. It is possible to collect such signals when *I*_pump_ is modulated in the frequency function, providing in this way a series of short laser pulses delivered to the investigated sample (Figure 5). That approach also provides information about the Kerr constant (*B*) value of the NLO active media. In the case of the p(SMER-AA) co-polymer, the photoisomerizations induced by UV light are of 1 order of magnitude lower Δ*n* intensity than in the case of p(SMER-SMA). Thus, it has to be underlined that thanks to the high structural order (due to the lower entropy resulting from the long aliphatic chains’ presence separating active side chains) in the p(SMER-SMA) co-polymer, the obtained NLO response was significantly higher and noticeable. The time constants describing the kinetics of the UV-induced molecular reorientations are equal to τ_dyn_^inc^= 27.7 ± 0.3 ms for the trans → cis transformation and τ_dyn_^dec^= 64.0 ± 2.0 ms for the signal reverse one (cis → trans) in the p(SMER-AA) material, respectively (Figure 5a). Meanwhile, the p(SMER-SMA) co-polymer photoisomerization changes are defined by τ_dyn_^inc^= 28.7 ± 0.3 ms for light controlled signal increase and τ_dyn_^dec^= 55.7 ± 1.2 ms for thermodynamic signal decay, respectively (Figure 5b). In here, it is visible that the dynamic part of the all-optical switching, which in our case relies on the same azobenzene fragment, is working with slightly the same kinetics defined by approximately the same time constant values.

Independently of the time constant values for both materials, the dynamic NLO response was still observed even for applied 500 Hz pump signal modulation, which is shown in Figure 5. The evaluated Kerr constant value is equal to 0.03 and 0.19 m/V^2^ (when the same experimental conditions were provided; *f* = 10 Hz and *I*_pump_ = 14.4 mW/cm^2^) for p(SMER-AA) and p(SMER-SMA), respectively.

To summarize the NLO, material, and kinetic parameters for the investigated photochromic polymers, all experimental data are gathered in Table 5. When discussing third-order NLO susceptibility values, they are slightly the same numbers, i.e. χ^(3)^ = 2.08 × 10^–6^ m^2^/V^2^ and χ^(3)^ = 1.64 × 10^–6^ m^2^/V^2^ for p(SMERe-AA) and p(SMERe-SMA), respectively. Such estimated parameters are consistent due to the literature values for similar photochromic polymers.^25^ As an example, considerably identical values of χ^(3)^ measured for analogous materials were observed recently. Photochromic co-polymers with the side chain built by NIPAM units gave third-order NLO susceptibility values in the range of (1.2–3.9) × 10^–5^ m^2^/V^2^, depending on the utilized aliphatic unit (methyl or ethyl) localized close to the azobenzene fragment.

**Table 5:** Nonlinear Optical and Material Parameters Together with Time Constant Values Defining Photoinduced Molecular Reorientations

parameter	p(SMERe-AA)	p(SMERe-SMA)
*d* (nm)	340	475
*T*_g_ (°C)	113	78
Δ*n*^a^	0.0423	0.0248
Δ*n*_max_	0.0674 *I*_pump_^max^= 31.4	0.0735 *I*_pump_^max^= 49.8
τ_inc1_^stat^ (s)	30.6 ± 0.8^b^	2.3 ± 0.1^a^
τ_inc2_^stat^ (s)	222.7 ± 20.0^b^	37.1 ± 0.3^a^
τ_dec1_^stat^ (s)	6.3 ± 0.3^b^	4.2 ± 0.2^a^
τ_dec2_^stat^ (s)	373.0 ± 114.7^b^	67.9 ± 5.4^a^
τ_inc_^dyn^ (ms)^a^^,^^c^	27.7 ± 0.3	28.7 ± 0.3
τ_dec_^dyn^ (ms)^a^^,^^c^	64.0 ± 2.0	55.7 ± 1.2
*n*_2_ (m^2^/W)	2.40 × 10^–4^	1.89 × 10^–4^
*n*_2_ (cm^2^/mW)	2.40 × 10^–3^	1.89 × 10^–3^
*χ*^(3)^ (m^2^/V^2^)	2.08 × 10^–6^	1.64 × 10^–6^
Δ*n*_dyn_^a^^,^^c^	4.18 × 10^–4^	2.67 × 10^–3^

a *I*_pump_ = 14.4 mW/cm^2^.

b *I*_pump_ = 18.0 mW/cm^2^.

c *f* = 10 Hz.

## 4 Conclusions

We have presented two photosensitive polymeric systems, which differ in their chemical construction. Slight structural changes introduced to their optically passive moieties resulted in significantly different and appealing NLO output, modulation, and signal stability. Starting from their synthesis method as well as transfer from the solid state toward the thin film shape, photochromic polymers were easy in fabrication and further manipulations. Performed spectroscopic experiments brought clear answer about the chemical structure influence (localized even in the passive and side-chain fragments) of the optical and nonlinear optical signals. We found that when a small and not chromophoric part is applied (p(SMER-AA)), the photoactive azobenzene groups together with main polymeric chains are in a high entropy state causing less available free volume inside the sample architecture, whereas the more ordered p(SMER-SMA) polymer provides quite regular and ordered space, which enables as much free space as needed to perform efficient, stable, and fast conformation phototransformations in the active region. It is easily visible when looking at kinetic parameters when time constant values of the photoinduced effect are at least 1 order of magnitude lower than in the case of the p(SMER-AA) polymer with its chaotic structure. Moreover, the photoinduced birefringence generated by the same *I*_pump_ value is 2 times higher if considering the p(SMER-SMA) system. Additionally, the Δ*n*_dyn_ value is 1 order of magnitude higher for the more ordered photochromic polymer, which indicates much faster and more efficient photoinduced conformational transformations. Achieved and estimated parameters like third-order NLO susceptibility or second NLO refractive index values are similar to each other and also to the other azobenzene-based photochromic macromolecular systems. Based on the achieved experimental results, it can be stated that by a simple and slight molecular change in the photosensitive materials, it is feasible to obtain highly responsive, stable, and repeatable organic materials. Thus, the presented approach to the materials engineering can pave the way for more efficient design and fabrication of the macromolecular photoresponsive components in the opto-electronic devices, such as optical logic gates, modulators, or reversible data storage.^4,5^

## Supporting Information Available

Description of synthesis, ^1^H NMR spectra; intramolecular hydrogen bond in the cis isomer of SMERe-acrylamide dimer (after geometry optimization) presentation; second-order kinetics of p(SMERe-AA) and p(SMERe-SMA): trans–cis photoisomerization (a) and cis–trans back transition (b) by spectrophotometry; and kinetics of the dynamic part of photoinduced birefringence (multiple trans–cis–trans conformational changes) for various signal modulation frequency (PDF)
