# Supplementary material for: Compatible Photochromic Systems for Opto-electronic Applications
Source: J Phys Chem B. 2021 Dec 4;125(49):13565–74. doi: 10.1021/acs.jpcb.1c08728 (PMC8686115; doi:10.1021/acs.jpcb.1c08728)

ELECTRONIC SUPPLEMENTARY INFORMATION

Compatible Photochromic Systems for Opto-Electronics Applications

Adam Szukalski, Aleksandra Korbut, Karolina Zieniewicz and Sonia Zielińska

Content:

S1.Synthesis of azocompounds

Table S1. Composition and conditions of the copolymerization processes.

Figure S1. ^{1}H NMR spectrum of azo copolymer p(SMERe-AA).

Figure S2. ^{1}H NMR spectrum of azo copolymer p(SMERe-SMA).

Figure S3. Intramolecular hydrogen bond in cis isomer of SMERe – acrylamide dimer (after geometry optimisation).

Figure S4. Second-order kinetics of p(SMERe-AA) and p(SMERe-SMA): trans-cis photoisomerization (a) and cis-trans back transition (b).

Figure S5. Kinetics of the dynamic part of photoinduced birefringence (multiple trans-cis-trans conformational changes) for p(SMERe-AA) (a-c) and p(SMERe-SMA) (d-f) for various signal modulation frequency: 50 Hz (a,d), 100 Hz (b, e) and 200 Hz (c, f), respectively. I_{pump}: 14.4 mW/cm^2.

S1. Synthesis of azocompounds.

All chemicals (sulfamerazine, 2-(N-ethylanilino)ethanol, azobisisobutyronitrile (AIBN, 98%), γ-butyrolactone, DMSO-d_6, methacrylic anhydride, 4-(dimethylamino)pyridine, 4-methoxyphenol, acrylamide (AA)) were purchased from a commercial source (Sigma Aldrich®), and were used without further purification. Only stearyl methacrylate (SMA) (Sigma Aldrich®) was purified from the inhibitor by washing with a 10% sodium hydroxide aqueous solution, and then with deionized water until neutralization. Afterwards, the SMA monomer was dried over anhydrous $MgSO_4$. Tetrahydrofurane, pyridine, sodium nitrite, anhydrous sodium acetate, sodium hydroxide, concentrated hydrochloric acid, and glacial acetic acid were purchased from POCH company (Poland).

The free radical polymerizations were carried out under inert, nitrogen atmosphere. Both monomers were used in the equimolar amounts and AIBN was used as an initiator. The reaction

mixtures were finally poured into 150 mL of cold water or methanol, whereupon the copolymers p(SMERe-SMA), and p(SMERe-AA) were precipitated, respectively. Afterwards, the resulting solids were filtered, washed with distilled water or methyl alcohol, and dried in 60 ˚C.

Table S1. Composition and conditions of the copolymerization processes.

	p(SMERe-AA)	p(SMERe-SMA)
M-SMERe	0.01 mol	0.01 mol
AA	0.01 mol	-
SMA	-	0.01 mol
AIBN	0.60 g	0.86 g
Solvent (v/v ratio)	30 ml of THF/water 9/1	30 ml of THF/γ-butyrolactone 7/3
Time of reaction	68 h	72 h
Temperature of reaction	70 ˚C	boiling point
Yield	67%	82%

Figure S1. ^{1}H NMR spectrum of azo copolymer p(SMERe-AA).

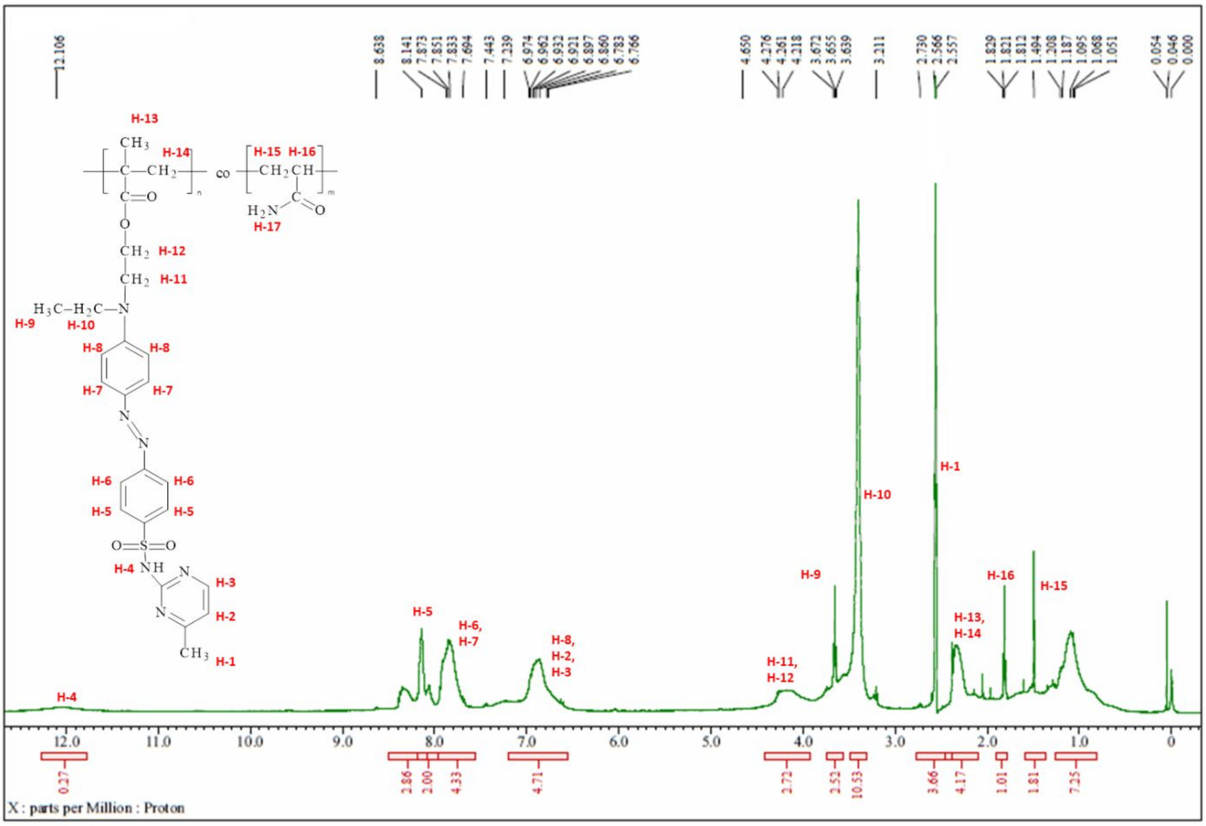

p(SMERe-AA): ^{1}H NMR (DMSO-d_6, with 0.05% v/v TMS, 400 Hz): δ_H ~12.06 (1H, s, H-4), ~8.14 (2H, s, H-5), ~7.84 (4H, s, H-6 and H-7), ~6.86-7.44 (5H, m, H-16, H-8, H-2 and H-3), ~4.65 (2H, m, H-12), ~4.26 (2H, m, H-11), ~3.66 (3H, m, H-9), ~3.21 (2H, m, H-10), ~2.56 (3H, s, H-1), ~2.35 (5H, m, H-14 and H-13), ~1.82 (1H, d, H-16), ~1.45 (2H, s, H-15).

Figure S2. ^{1}H NMR spectrum of azo copolymer p(SMERe-SMA).

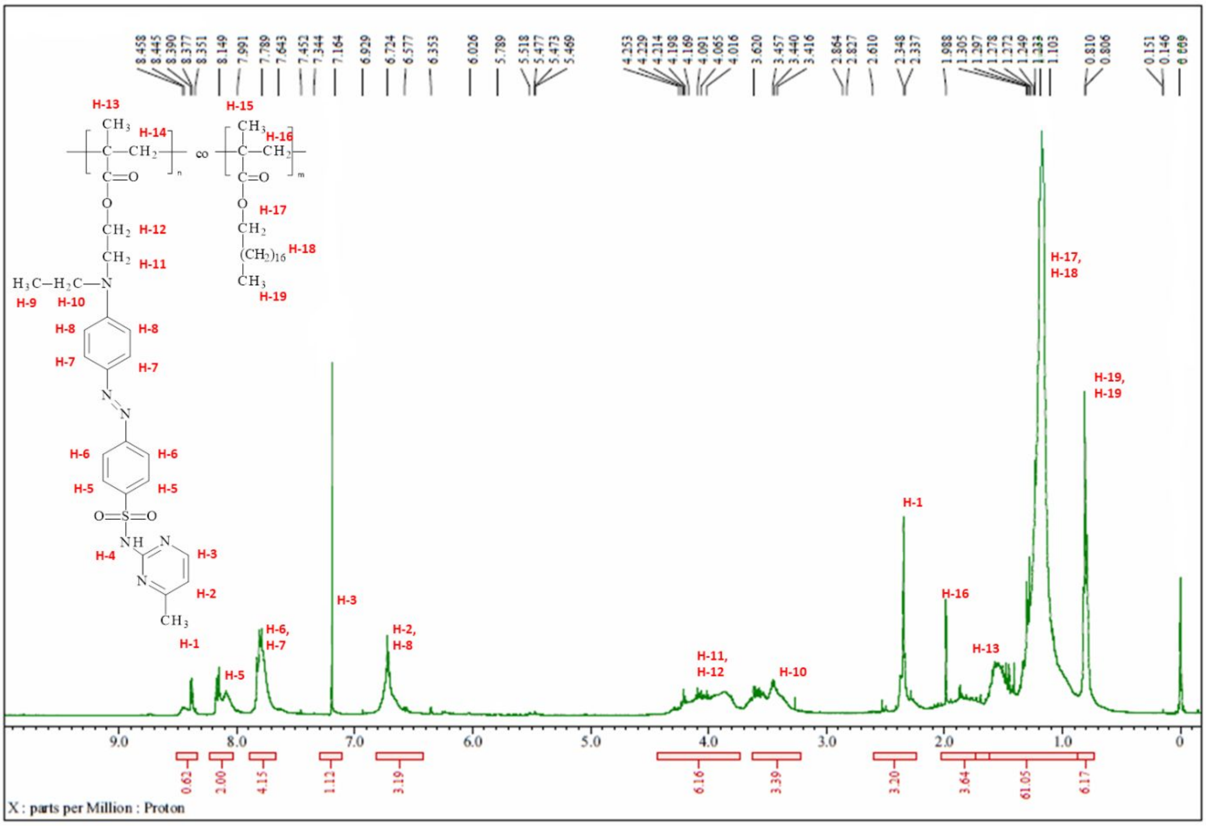

p(SMERe-SMA): ^{1}H NMR ($CDCl_3$-d_6, with 0.05% v/v TMS, 400 Hz): δ_H ~8.15 (2H, s, H-5), ~7.79 (4H, s, H-6 and H-7), ~7.16 (1H, s, H-3), ~6.93 (3H, s, H-8 and H-2), ~4.02-4.23 (4H, m, H-11 and H-12), ~3.44-3.62 (3H, m, H-10), ~2.61 (3H, s, H-1), ~1.98 (3H, s, H-16), ~1.27-1.31 (3H, m, H-13), ~1.10-1.25 (34H, m, H-17 and H-18), ~0.81 (6H, d, H-9 and H-19).

Figure S3. Intramolecular hydrogen bond in cis isomer of SMERe – acrylamide dimer (after geometry optimisation).

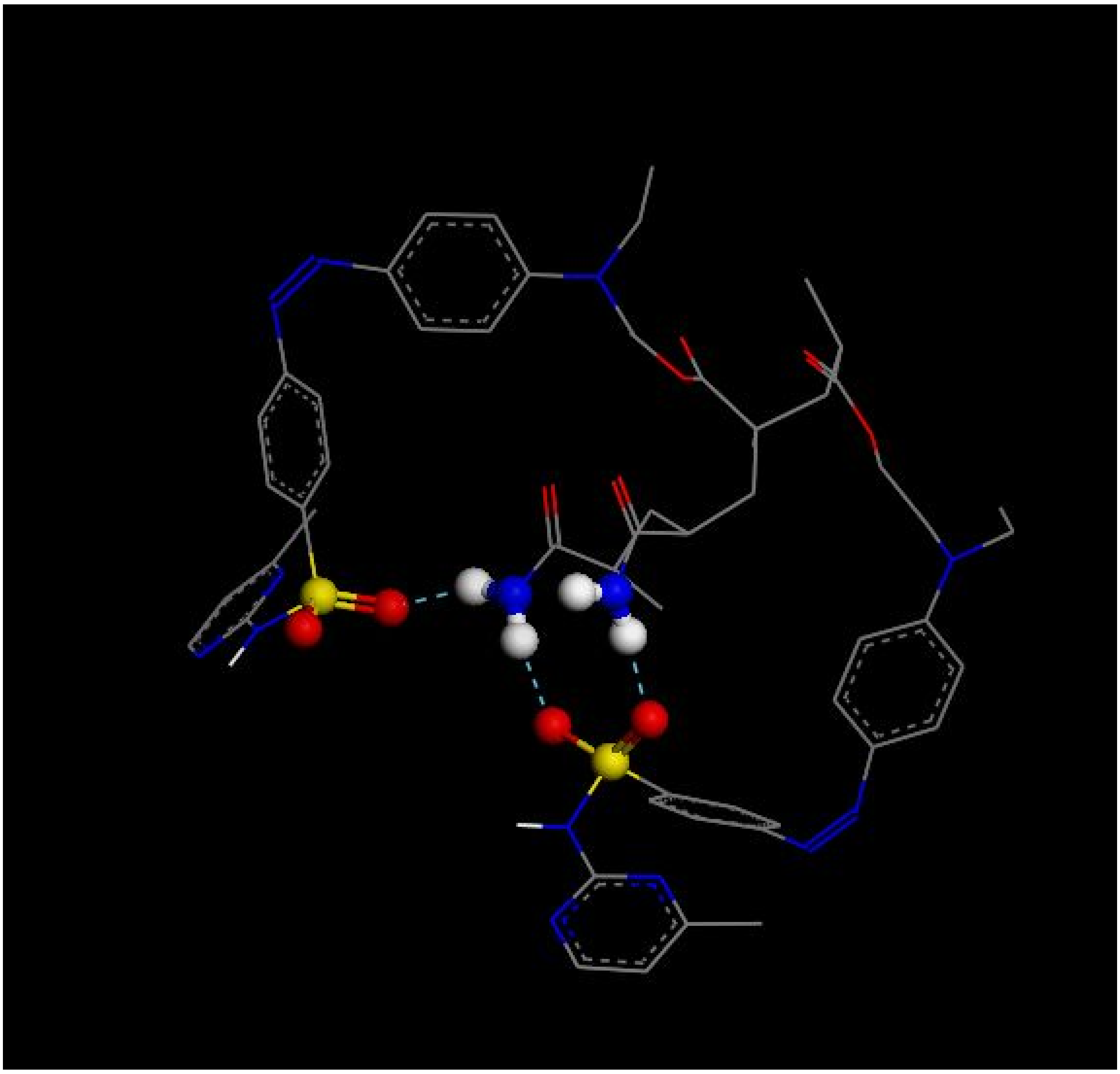

Figure S4. Second-order kinetics of p(SMERe-AA) and p(SMERe-SMA): trans-cis photoisomerization (a) and cis-trans back transition (b).

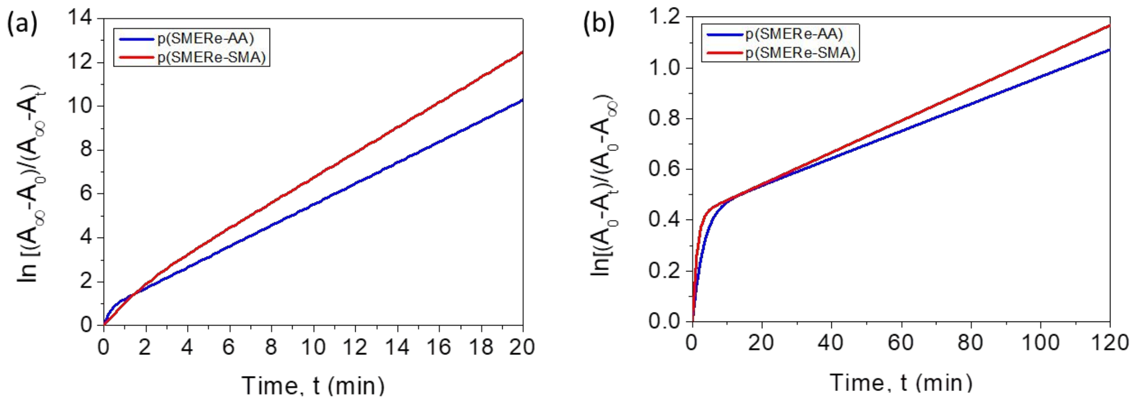

Figure S5. Kinetics of the dynamic part of photoinduced birefringence (multiple trans-cis-trans conformational changes) for p(SMERe-AA) (a-c) and p(SMERe-SMA) (d-f) for various signal modulation frequency: 50 Hz (a,d), 100 Hz (b, e) and 200 Hz (c, f), respectively. I_{pump}: 14.4 mW/cm^2.

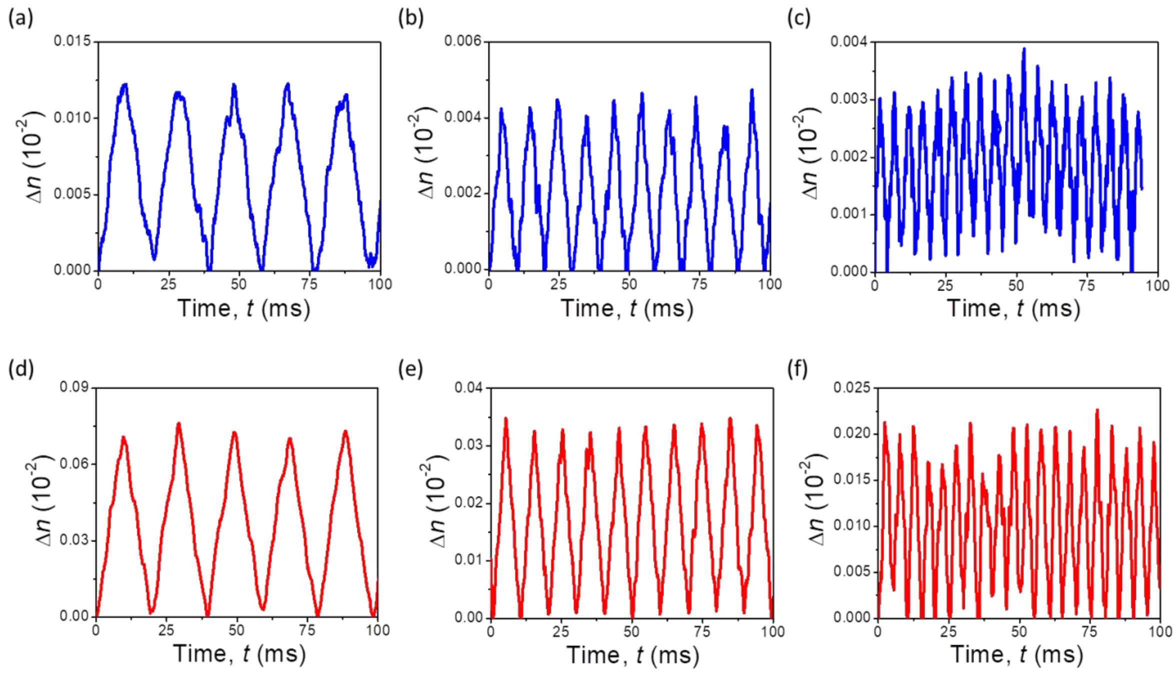

Supplement: Supplementary file 1 — jp1c08728_si_001.pdf [file jp1c08728_si_001.pdf]
